# Circulating Contact-Pathway-Activating Microparticles Together with Factors IXa and XIa Induce Spontaneous Clotting in Plasma of Hematology and Cardiologic Patients

**DOI:** 10.1371/journal.pone.0087692

**Published:** 2014-01-31

**Authors:** Elena Lipets, Olga Vlasova, Evdokiya Urnova, Oleg Margolin, Anna Soloveva, Olga Ostapushchenko, John Andersen, Fazoil Ataullakhanov, Mikhail Panteleev

**Affiliations:** 1 Federal Research and Clinical Center of Pediatric Hematology, Oncology and Immunology, Moscow, Russia; 2 HemaCore LLC, Moscow, Russia; 3 Lomonosov Moscow State University, Moscow, Russia; 4 National Research Center for Hematology, Health Ministry RF, Moscow, Russia; 5 National Institute of Allergy and Infectious Diseases, Rockville, Maryland, United States of America; 6 Center for Theoretical Problems of Physico-Chemical Pharmacology, Russian Academy of Sciences, Moscow, Russia; University of Bonn, Institut of experimental hematology and transfusion medicine, Germany

## Abstract

**Background and Objective:**

Using an in vitro experimental model of immobilized tissue factor-initiated clot growth in platelet-free plasma (thrombodynamics), we observed formation of activator-independent isolated spontaneous clots (SC) throughout the plasma volume in patients with cardiac infarction, acute leukemia, hemolytic anemia, and some other disorders. The aim of this work was to characterize this phenomenon and to identify the mechanisms of SC formation.

**Methods and Results:**

Tissue factor inhibitor (VIIai) prevented SC in only 2 out of 23 patient plasma samples. Specific inhibitors of factors IXa and XIa were efficient in all 8 cases that we tested. Also, only factors IXa and XIa added to normal donors’ plasma induced SC formations from isolated centers, in a pattern similar to that in patients’ plasma. In contrast, factors VIIa, Va, tissue factor induced uniform plasma clotting. SC disappeared after high-speed centrifugation. However, phospholipid supplementation of centrifuged plasma returned them at least partially in 5 out of 22 patients’ plasmas, indicating some other role of microparticles than providing phospholipid surface. Circulating procoagulant microparticles isolated from plasma directly activated factor XII in buffer and in diluted plasma. Flow cytometry revealed an increase in procoagulant microparticles in patients’ plasmas with SC.

**Conclusion:**

Our data suggest that combination of circulating active factors (specifically, factors IXa and XIa) with circulating procoagulant and contact-pathway-activating microparticles is the predominant mechanism causing spontaneous clotting in patient plasma.

## Introduction

A number of hematological and cardiovascular disorders is associated with the presence of procoagulant material in the patients' blood, such as: active coagulation factors [1]–[6]; procoagulant microparticles shed by tumor cells [7], blood cells[8]–[10], or by endothelium [11]; tissue factor (TF) that may appear on the endothelium and monocytes following stimulation by lipopolysaccharides, TNF, IL1 [12], elevated C-reactive protein [13]. The functional importance of this procoagulant material in the majority of cases is unclear. Its characterization using homogeneous blood coagulation assays is difficult because of the addition of a potent external activator and of the mixing with this activator. In the spatially non-uniform clot growth assays [14], [15], the activator is localized on the surface, so that contributions of procoagulant material that accelerate coagulation and material that activate coagulation itself can be clearly distinguished. The material able to accelerate reactions only would increase clot growth rate from immobilized activator, while the one capable of clotting activation would also induce spontaneous clotting (SC). Using this approach, we detected SC in the plasma of patients with a number of hematological disorders. This is a pilot study devoted to this phenomenon. So we tried to identify some common mechanisms of SC that could be independent of clinical course details of a definite patient. Mechanisms leading to SC in certain diseases cases need further investigation. Our results show that circulating microparticles activate coagulation through the contact pathway and cause SC. Though causes of this clotting can be different in different disorders, increase of microparticles number or long-lived active coagulation factors' concentrations (specifically, factors IXa and XIa) feasibly explain spontaneous clotting in the overwhelming majority of the cases that we have observed.

## Materials and Methods

### Materials

The following materials were obtained from the sources shown in parentheses: thromboplastin (TF), kaolin (Renam, Moscow, Russia), fVIIa (Novo Nordisk, Bagsvaerd, Denmark) fVa, fIXa, fXIa, fV, fIX, Anti-Human Factor XI antibody (Haematologic Technologies Inc., Vermont, USA), fII (Enzyme Research Laboratories Inc, South Bend, IN, USA), fVIII (Hemophil M, Baxter, Moscow, Russia), L-α-phosphatidylserine (Brain, Porcine) (PS), L-α-phosphatidylcholine (Egg, Chicken) (PC) (Avanti Polar Lipids, Alabaster, Alabama, USA), specific fXIIa inhibitor corn trypsin inhibitor (CTI) (Institute of Protein Research, Russian Academy of Sciences, Pushchino, Russia), D-phenylalanyl-L-prolyl-L-arginine chloromethyl ketone (PPACK), (Calbiochem, Emd Biosciences Inc, San Diego, CA, USA), mouse anti-human CD61 labeled with peridinin-chlorophyll-protein complex (anti-CD61-PerCP), annexin V-FITC (BD Biosciences, San Jose, CA, USA), chromogenic substrate for fXIIa, fXIa and kallikrein S2302 (CHROMOGENIX, Instrumentation Laboratory company, Bedford, MA, USA), fluorogenic substrate for thrombin Z-Gly-Gly-Arg-AMC·HCI (Bachem Americas, Inc., Torrance, CA, USA). Nitrophorin 2 was prepared as described [16]. All other reagents were obtained from Sigma-Aldrich (St. Louis, Missouri, USA).

### Subjects

The experiments were carried out on plasmas obtained from healthy donors and plasmas from patients suffering from various conditions. A list of study subjects with their diagnoses, ages, and coagulation tests data can be found in Tables S1–S4.

### Ethics Statement

All patients gave their written informed consent, and the study protocol was approved by the Ethics Committee of the Center for Theoretical Problems of Physicochemical Pharmacology.

### Preparation of Plasma Samples

Blood samples were collected in tubes containing 3.8% citrate. Whole blood samples were stored for not more than one hour. After 15 min centrifugation at 1600 g, the upper two-thirds of the supernatant were collected, and then platelet-free plasma (PFP) was obtained by centrifugation for 5 min at 10000 g. Some of the experiments were carried out with thawed plasma. For thawing, plasma samples were placed in a water bath at 37°C and incubated for one hour. Centrifugation for 30 min at 16000 g was used to remove microparticles from PFP.

### Thrombodynamics

**Figure 1:**
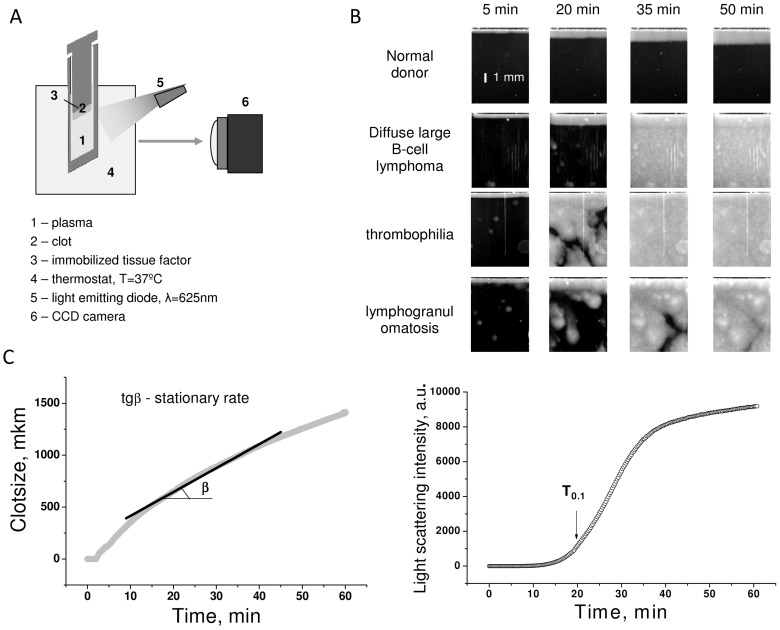
Spontaneous clotting during the thrombodynamics assay. (A) Experimental design. Plasma was placed into a microchamber and put in contact with tissue factor covered surface. Light scattering signal from growing clot was recorded on CCD camera. (B) Images of clot growth in normal plasma and in patient’s plasma. Activating surface is in the bottom of the images, light band above is a growing clot, light areas that appear throughout the volume are spontaneous clots. (C) Determination of clot growth rate and T_0.1_ time. 10 to 40 minutes region of clot size versus time plot was approximated with line. Stationary clot growth rate was determined as slope of this line. T_0.1_ was determined as time when mean light scattering of plasma volume excluding clot from activator and microchamber walls reaches 0.1 of maximal level.

For experiments on spatial clot growth, 150 µL of plasma were supplemented with 6 µL of CTI solution (5 mg/mL), incubated for 10 minutes at 37°C, recalcified with 3 µL of CaCl_2_ (1 M solution), and put into a polysterene microchamber (HemaCore, Moscow, Russia). The microchamber was placed into a thermostat, and an insert with immobilized tissue factor was immersed into the microchamber. Clot growth began from the tissue factor-covered surface. It was monitored by light scattering using a digital camera. Clot images were used to determine the clot size versus time dependence. Stationary clot growth rate was determined as a linear approximation of this curve in the period from 10 to 40 minutes after the beginning of clot growth. To characterize spontaneous clotting intensity, we selected a maximum area in the image excluding the activator-induced clot and the microchamber walls, and calculated the average light scattering – time relationship in it. The time when intensity reached 0.1 of maximum (T_0.1_) was used as quantitative parameter of spontaneous clotting (Figure 1). Activators with immobilized TF for the Thrombodynamics assay prepared essentially according to the technique described in [17] were provided by HemaCore. CTI was dissolved in the 0.75 M HEPES, pH = 7.4 buffer. The buffer was used at a high concentration to stabilize the pH value within the 7.2–7.6 range.

### Thrombin Generation Assay

PFP was mixed with fluorogenic substrate for thrombin Z-Gly-Gly-Arg-AMC•HCI and HEPES buffer for pH stabilization. Final concentrations are 400 µM and 20 mM respectively. 100 µl of the mixture was placed per well of a flat-bottom 96-well plate. Thrombin generation was started with 20 µl of solution contaning 100 mM CaCl2 or 100 mM CaCl2 and 30 pM TF. Additional calibrating wells were supplemented with 20 µl of the substrate cleavage product (AMC) solution or 20 µl of buffer. Each sample was reproduced in duplicates. AMC fluorescence was excited at 390 nM and measured at 460 nM with an Appliskan device (Thermo Scientific Ins, Waltham, Massachusetts, USA). Data analysis was carried out using Origin 8.0 software (Microcal Software, Northampton, MA, USA). Fluorescence was converted to AMC concentration with the help of a calibration determined individually for each specimen by measuring difference between fluorescence of a sample with known AMC concentration and with buffer. Nonlinear fluorescence dependence on AMC concentration was measured in preparatory experiments and it was taken into account for AMC concentration calculation in a sample. Thrombin concentration was obtained using the previously measured kinetic constants for a given fluorescence substrate (KM = 156 µM; kcat = 46 min^−1^).

### Preparation of fVIIai

The fVIIa inactivation method was carried out as described in [18] with modifications. FVIIa was incubated with PPACK for 60 min at 4°C at a 1∶ 2 molar ratio. The obtained fVIIai was separated from PPACK by dialysis against Tris buffer. The final concentration of fVIIai was measured on a spectrophotometer by absorption at 280 nm. 50 nM fVIIai completely suppressed clotting caused by 0,25 pM TF for 2 hours of experiment. Complete inactivation of fVIIa was checked in a chromogenic assay. FVIIai sample and calibration fVIIa samples 10÷1,25 pM dissolved in Ca solution were incubated 5 min with TF, then mixed with fX and incubated 15 min. The reaction was stopped by EDTA addition and amount of gained fXa was evaluated by substrate S 2765 cleavage rate. PPACK removal was proved by the fact that fVIIai solution had no effect on fluorogenic substrate cleavage by thrombin.

### Preparation of Phosholipid Vesicles

Artificial phospholipid vesicles composed of 80% PC and 20% PS were prepared according to the protocol recommended by Avanti Polar Lipids with minor changes. Phospholipids were transferred with a Hamilton syringe into a round-bottomed flask, dried for 30 minutes under a nitrogen current to eliminate chloroform, and hydrated in the 20 mM HEPES, 140 mM NaCl, pH = 7.5 buffer for 30 minutes at T>50°C. The flask was fixed upon a shaker. The resulting solution was treated with a freeze – thaw cycle, heated to T>50°C, and forced through the extruder membrane. The pore diameter was 100 nm.

### Flow Cytometry

Microparticles in plasma were measured using a Becton Dickinson FACSCalibur flow cytometer (San Jose, CA, USA) with CellQuest software (Becton Dickinson) according to the technique described in [19], with modifications. A 200 µL platelet-free plasma sample was centrifuged for 30 minutes at 16000 g, 180 µL of the plasma were removed. The remaining 20 µL were resuspended in 180 µL of the buffer A (150 mM NaCl, 2.7 mM KCl, 1 mM MgCl_2_, 0.4 mM NaH_2_PO_4_, 20 mM HEPES, 5 mM glucose, 0.5% bovine serum albumin, pH 7.4, filtered through a 0.22 µm membrane) containing 0.38% of citrate. After that, the sample was centrifuged again for 30 min at 16000 g, and 180 µL of the supernatant were removed.

The suspension with the obtained microparticles at 5 µL was supplemented with 35 µL of buffer A with CaCl_2_ (10 mM) (or without Ca in the EDTA control), 5 µL of 10% annexinV-FITC, and 5 µL of PerCP-labeled anti CD61 Mab, then incubation for 15 minutes at room temperature followed. Prior to the measurement, 200 µL of the buffer A with CaCl_2_ (2.5 mM) (or without Ca in the EDTA control) were added. Cytometer measurements were performed for 1 min at the minimal flow rate. Fluorescence cannel FL1 was chosen as the primary one. Count Bright calibration beads (Life Technologies, Carlsbad, CA, USA) were used for quantitative assessment of the microvesicles concentration in the sample. The sample concentration was multiplied by a 250/5/10 coefficient to obtain the plasma concentration of vesicles. The FL1 gating region was selected by using experiments with addition of 5 µL of EDTA 100 mM with stains, the FL3 border was obtained in a no-antibody experiment. The FSC upper boundary of microparticles region was the lower bound of platelets region.

### Contact Pathway Factors Activity Measurement

Activity of contact pathway factors was estimated by the cleavage rate of S2302. Absorption at wavelength 405 nm from the substrate cleavage product was measured with an Appliskan device (Thermo Scientific Ins, Waltham, Massachusetts, USA). Substrate cleavage rate was used to evaluate contact pathway factors activity. CTI addition showed what part of a signal was produced by fXIIa. An 8 µL volume of the S2302 (2 mM) substrate and 92 µL of the sample were transferred into a microplate well. The samples were prepared in the following manner: 1 mL of PFP was centrifuged for 30 min at 16000 g, the upper 90% portion was microparticle poor plasma, the lower 10% portion was resuspended in 900 µL of buffer A with citrate; centrifugation was repeated and 90% of the supernatant was eliminated. After that, the microparticles concentrate was either diluted fivefold with the buffer A containing citrate or washed two more times with the buffer A and diluted fivefold. The following samples were measured for each donor: microparticles after three washes; microparticles-poor plasma diluted 50-fold, microparticles after the first wash (containing plasma diluted 50-fold); microparticles after the first wash with CTI added (final concentration, 200 µg/mL); microparticles after three washes with fXII added (final concentration, 22 nM); buffer; buffer with 22 nM fXII added.

*Statistics.* In the Results section, means ± standard deviation are presented except for the data of chromogenic assay where are presented means ± standard errors of the mean. The difference between groups was considered significant at p<0.05 according to Wilcoxon sign rang test with Bonferroni correction for multiple comparisons.

## Results

### Spontaneous Clotting Occurrence in Various Diseases and its Reproducibility

In Thrombodynamics assay, external activation is by tissue factor localized on a surface. Accordingly, in normal plasma, we observed clotting propagating from this surface only (Fig. 1). In patients’ plasma with various diseases, we frequently observed clotting far from the activator that implied existence of some activating material in plasma itself (Fig. 1). Among the persons who did not receive anticoagulant therapy, spontaneous clotting occurred in 4 out of 12 patients with autoimmune hemolytic anemia, in 2 out of 5 subjects with acute leukemia, in 9 out of 14 individuals with myocardial infarction, and in 1 out of 35 healthy donors (Table 1). Therefore, spontaneous clotting (SC) was significantly more common in diseased subjects than in healthy ones.

**Table 1:** Spontaneous clotting occurrence in plasma of patients with various disorders.

Diagnosis	Number of patients	Number of patients with SC	%
Autoimmune hemolytic anemia	12	4	33
Cardiac infarction	14	9	64
Acute leukemia	5	2	40
Healthy controls	35	1	2.9

The phenomenon was reproduced when the test was repeated on the same plasma sample with SC, and the T_0.1_ coefficient of variation was 11±12% (n = 12). After a plasma freeze/thaw cycle, spontaneous clotting persisted as well, the mean T_0.1_ change was −10±60% (n = 7). Therefore, the SC changes with freezing could vary between samples, but no qualitative systemic shift occurred, which implies that thawed plasma could be used for analysis of this phenomenon. Finally, if platelet-free plasma was kept at room temperature for 3 hours, T_0.1_ changed by 8±11% (n = 3). Storage for 5–7 hours sometimes resulted in slightly decreased clotting intensity, but the phenomenon still persisted.

One could presume that the cause of spontaneous clotting is that the amount of the added contact pathway inhibitor, CTI, is insufficient for complete inhibition of the activation resulting from the contact with the microchamber walls. In this case, the increasing surface area to microchamber volume ratio should have resulted in a reduction of the T_0.1_. Experiments with three SC plasma samples demonstrated that an increase in the microchamber width from 0.85 mm to 2.4 mm entailed no significant change in the T_0.1_ (data not shown), and activation occurs in the plasma volume.

**Figure 2:**
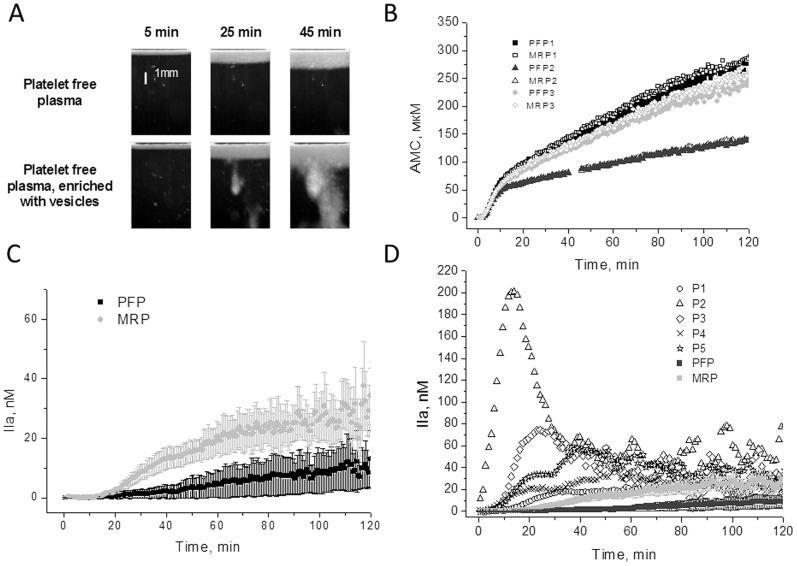
Plasma samples with SC in thrombodinamics and in thrombin generation assays. (A) Photographs of clot growth in normal donor platelet free plasma and in plasma enriched with vesicles 3.3-fold. (B) AMC generation curves in PFP (filled symbols) and microparticles rich plasma (MRP) (empty symbols) of 3 normal donors activated by 5 pM TF. (C) Average±se thrombin generation curves in normal donors’ platelet free plasma (PFP) and MRP for 3 donors. (D) Thrombin generation curves for 6 patients with SC in thrombodinamics assay and average thrombin generation curves in normal donors PFP and MRP. In C and D thrombin generation was started with racalcification. No external activator was added.

It was desirable to confirm significance of the SC phenomenon with an independent method, preferrably a widely used one, for example with thrombin generation test. For this purpose, we compared thrombin generation in PFP of normal donors with PFP of normal donors enriched with microparticles (the lower 30% of plasma after centrifugation for 30 minutes at 16000 g) as a model of SC (Fig. 2A) and with PFP of patients having natural SC. Thrombin generation activated with 5 pM TF was identical in PFP and PFP enriched with microparticles (MRP) of the same donor, which could be explained by high level of activation throughout the sample volume in this assay (n = 3) (Fig. 2B). The most clearly it could be seen on AMC generation curves. But when we carried out the same test with recalcification only without TF MRP was clearly distinct (n = 3) (Fig. 2C). Thrombin generation in patients' PFP were even greater than in MRP or on the similar level (n = 5) (Fig. 2D). Information about the patients one can find in Table S1.

### The Role of Different Active Facttors in Spontaneous Clotting

**Figure 3:**
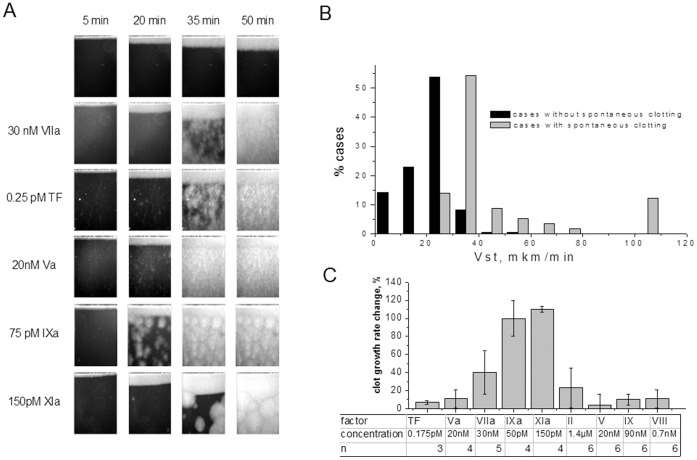
Active factors influence on thrombodinamics. (A) Spontaneous clotting patterns after active factors addition. (B) Clot growth rate distributions in patients’ plasma with SC and without. (C) Relative clot growth rate change after active factors and procoagulant zymogens addition to normal donors’ plasma. Concentrations of supplemented factors and number of donors are shown in table below the histograme.

Active factors are known to circulate in patients’ plasma in some medical conditions [3]–[5], [20], [21]. To find out whether they can induce spontaneous clotting in the Thrombodynamics assay, we compared test results obtained for patients’ plasmas and plasmas of healthy donors supplemented with factors that have long plasma lifetimes (comparable with time between venipuncture and beginning of a test): fVa, fVIIa, soluble TF, fIXa, and fXIa [22]–[24]. Addition of fVa and fVIIa resulted in uniform clotting throughout the plasma volume, which is untypical for patient plasma. FIXa and fXIa, on the contrary led to distinct clotting centers' appearance, as is the case with most clinical samples (Fig. 3A).

SC associated with increased stationary rates in the overwhelming majority of patient plasmas. Figure 3B shows the stationary rate distribution for experiments with and without SC. Among fVa, fVIIa, soluble TF, fIXa, and fXIa, only the latter two, along with SC initiation, had a marked, statistically significant effect on the stationary rate (Fig. 3C).

Furthermore, in contrast to other stable clotting factors, fIXa and fXIa caused SC and clot gowth rate increase in low concentrations that were fractions of percent of their zymogen physiological concentration. These are concentrations that could be expected to be found in patients' plasma. FVIIa and fVa require concentrations comparable to their zymogen physiological concentration to cause SC (Fig 3C), which are by orders of magnitude greater than those that can circulate in vivo.

**Figure 4:**
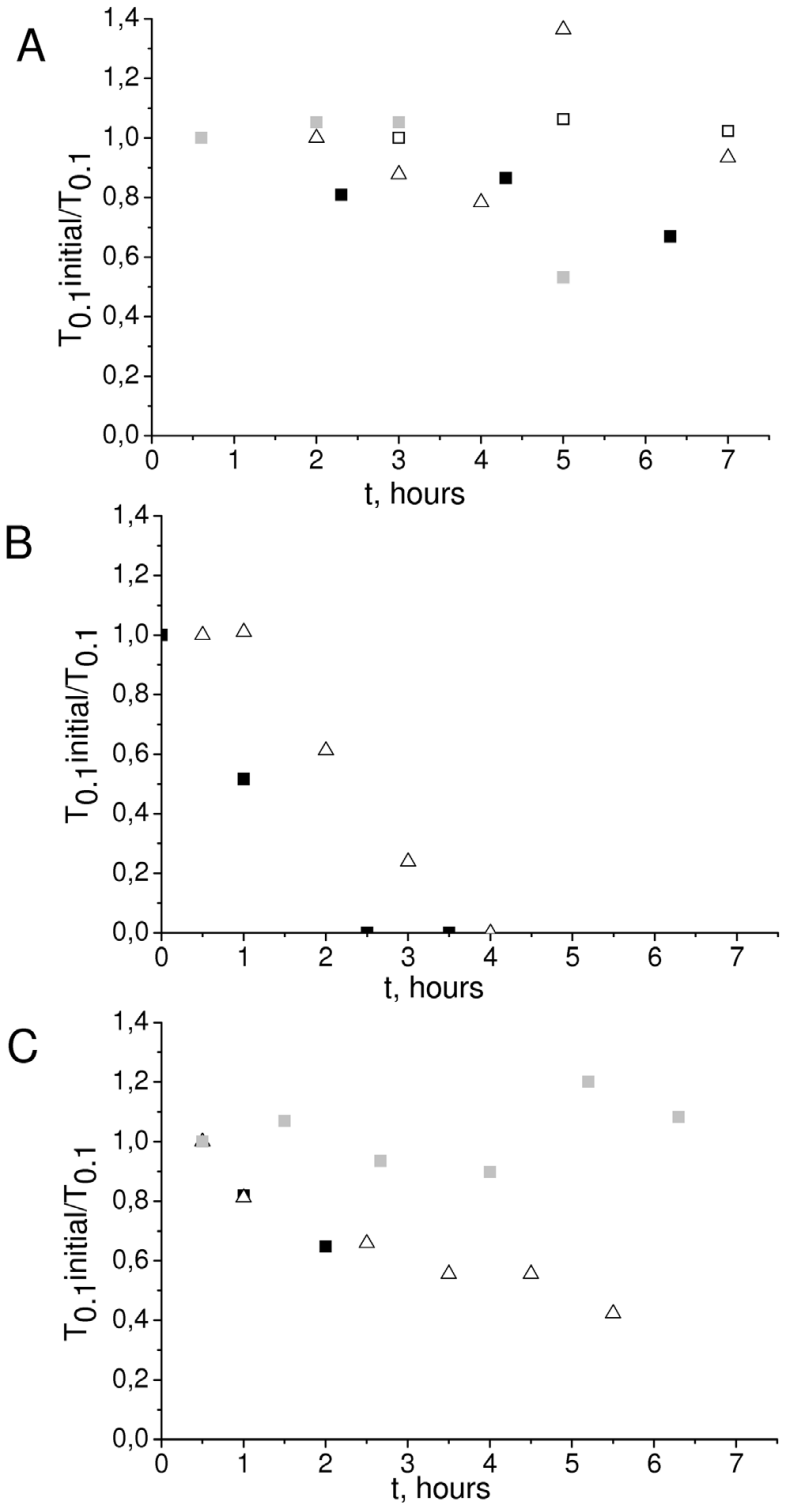
Stability of natural SC and SC induced by addition of factors IXa and Xia. Dependence 1/T_0.1_ of storage time in plasma from four patients. Different symbols correspond to different patients (A). The same dependence in healthy donors’ plasma with addition of 75 pM of fIXa (B) and 150 pM of fXIa (C). T_0.1_ is normalized to the T_0.1_ first-point plasma value.

Spontaneous clots persisted in patient plasma after its storage for 3 hours or longer (Fig. 4A). It was unclear whether fIXa and fXIa could retain their activity over this time. We compared stability of the SC phenomenon induced by the addition of these factors to healthy donor plasma with the stability data obtained for the patient plasmas. SC in a sample supplemented with 75 PM of fIXa that usually induce clotting within 30 to 40 min disappeared within 3 hours (Fig. 4B); SC in samples supplemented with 150 pM of fXIa were more stable (Fig. 4C), but these still disappeared earlier than in patients. These data suggest that fIXa and fXIa play a role in the formation of spontaneous clots, but they seem to be insufficient to explain the stable SC under plasma storage in all cases. Active factors should be protected from inhibition or to be constantly generated.

**Figure 5:**
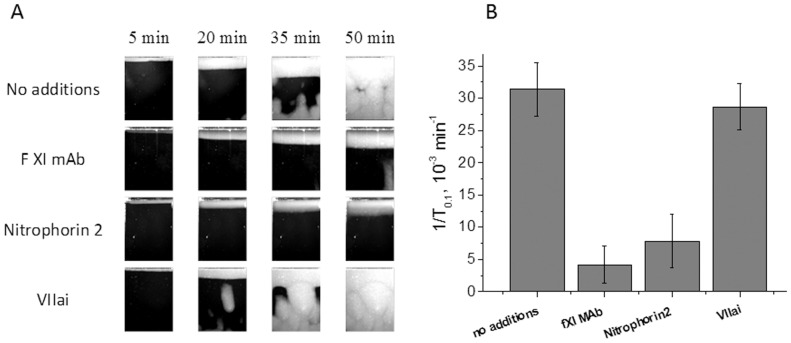
The role of TF, factors IXa, and XIa in spontaneous clotting. (A) Clot growth images from thrombodynamics assay in four versions: a patient PFP without any inhibitors, the same plasma after addition of 0.1 mg/ml inhibiting monoclonal antibody to fXI, after addition of 10 nM Nitrophorin2, fIX inhibitor, after addition of 50 nM VIIai, TF inhibitor. (B) Mean 1/T_0.1_±se in plasma samples of eight patients according to described four experiment versions.

Factors that seemed to be feasible reason for SC according to experiments with modeled SC we tried to inhibit in plasma with natural SC. We also tried to inhibit TF as it is considered to be essential factor of hypercoagulation at different diseases in literature. We supplemented patients’ plasma with tissue factor inhibitor, VIIai (n = 23), fXIa inhibitor, fXI mAb (n = 8), fIXa inhibitor, Nitrophorin2 (n = 8). The information about the patients is given in table S2. Figure 5A demonstrates characteristic clotting images seen in inhibitor-free plasma and plasma supplemented with fXI mAb (0.1 mg/ml), Nitrophorin2 (100 nM), VIIai (50 nM). The mean 1/T0.1 of 8 samples for each test version is represented in Figure 5B. TF inhibition was essential in 4 of 23 samples. In 2 of these samples (a patient with acute leukemia and a patient with liver cancer and ilio-femoral venous thrombosis) VIIai eliminated SC completely. FIXa and fXIa inhibition prevented SC or strongly reduced them in all the observed cases.

Therefore, SC may occur in plasma as a result of the TF presence, but, in most cases, the cause is different. FIXa and fXIa are feasible reason for SC but their presence along cannot explain SC stability in some cases and clotting from the separate centers.

### The Role of Zymogens in Spontaneous Clotting

An alternative explanation of the SC phenomenon is that patient plasma does not contain additional activating material compared with the plasma of healthy donors, but has a lower activation threshold instead. In this case, contact activation always present in experiments in vitro but too weak to pass the normal plasma threshold could trigger coagulation in patient plasma and cause SC. We supplemented plasmas of healthy donors with procoagulant zymogens that we expected to affect this threshold based on previous reports [25] (fII and fV), as well as those not expected to have an effect (fVIII and fIX). Doubling of the healthy donor plasma concentrations of these zymogens caused no spontaneous clotting (data not shown) or significant clot growth rate increase (Fig. 3C). These data do not support hypothesis that SC are caused by the threshold decrease.

### The Role of Microparticles in Spontaneous Clotting

It would be natural to expect spatially uniform clotting from an enzyme uniformly distributed in plasma. The rather small number of the SC centers may be explained by the fact that active factors bind to less frequently occurring larger particles and their surface triggers coagulation faster. Phospholipid vesicles may naturally serve as such particles.

**Figure 6:**
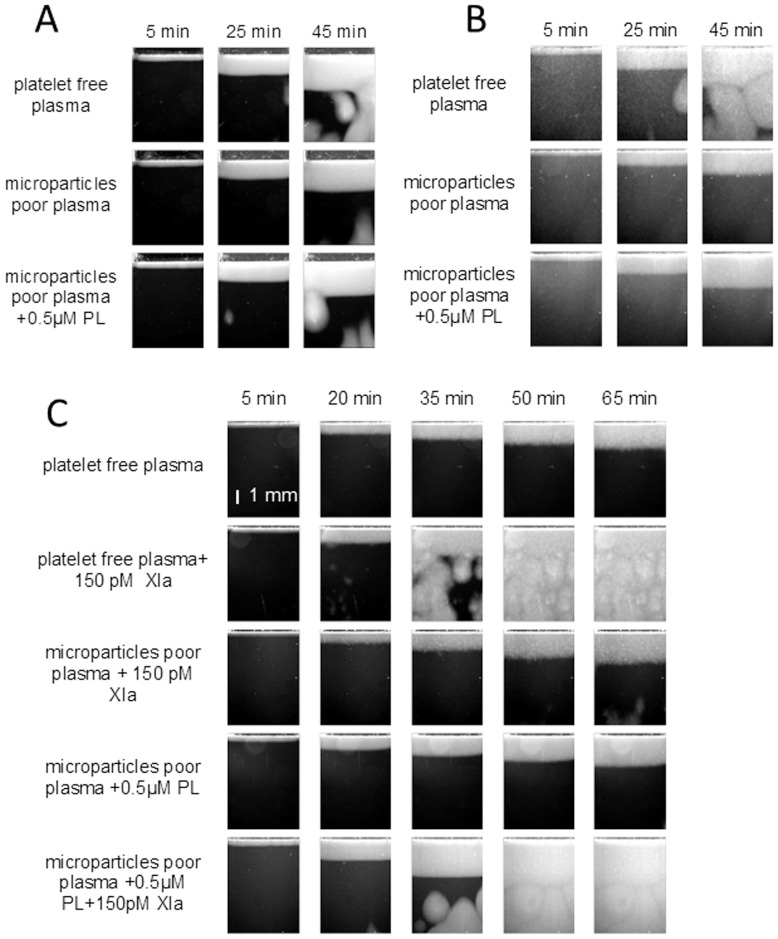
The role of microparticles and phospholipid surface in spontaneous clotting. Representative patterns of clotting in plasma cleared from microparticles by centrifugation 30(A) (n = 5) or not(B) (n = 17) and in normal plasma supplemented with fXIa (C).

Centrifugation of normal PFP supplemented either with fIXa or fXIa for 30 minutes at 16000 g eliminated SC in the middle layer (40%) of plasma volume or made SC considerably rarer and more uniform (Fig. 6C). SC were not formed in the upper layer (30%) either, and the lower layer (30%) formed clots much faster than PFP did. Clotting in the different layers of patient plasma behaved in a similar manner.

The most important factor for SC could be the phospholipid microparticle membrane or some other component. We replenished the middle 40% (n = 12) or upper 90% (n = 10) layer of patient plasma (Table S3) centrifuged at 16000 g with artificial phospholipid vesicles at a concentration of 0.5 µM. The two ways of plasma preparation have not shown qualitative difference so the results are represeted for the joined group. The 0.5 µM concentration of phospholipids was enough to bring clot growth rate in plasma from middle 40% layer roughly equal to that in PFP. Under the present centrifugation regime microparticles are not removed from upper layers complitly so we considered the spontaneous clotting pattern not to recover if the number of SC centers decreased more than twice. In patient plasmas following the addition of vesicles the clot growth rates increased but the spontaneous clotting patterns was recovered in 5 (Fig. 6A) (No. 2, 6, 11, 14, 20 in Table S3) plasma samples and was not in the other 17 (Fig. 6B). The number of centers in model SC induced by addition of fIXa or fXIa to normal PFP redused considerably also (Fig. 6C).

**Figure 7:**
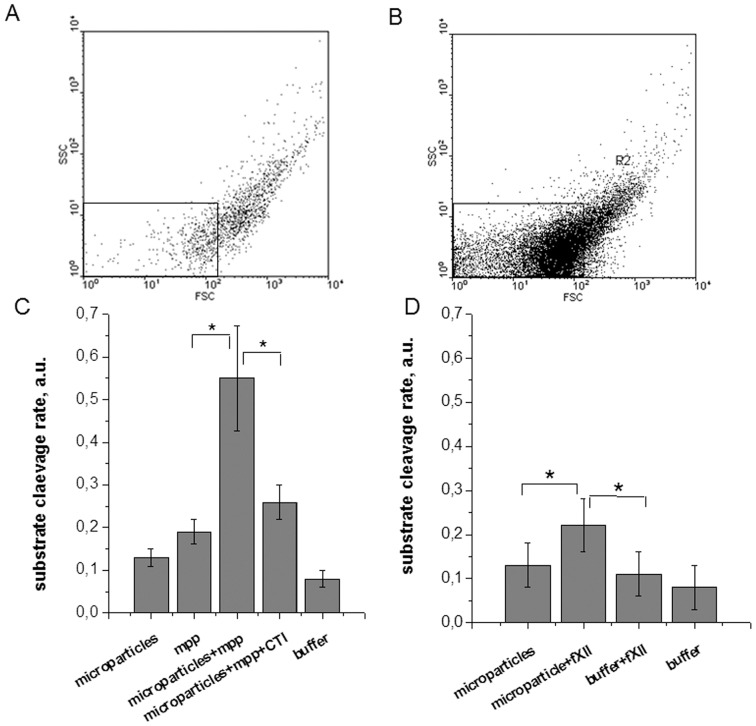
Contact pathway activation by microparticles. (A,B) Representative dot plots of microparticle samples from (A) normal donor and (B) patient plasmas. The rectangle is a microparticle region. It’ upper FCS bound is chosen at the lower bound of platelet region. (C,D) Substrate S2302 cleavage rate in following samples: (C) microparticles after three washes; microparticles-poor plasma diluted 50-fold (mpp), microparticles after the first wash (containing plasma diluted 50-fold); microparticles after the first wash with CTI added (final concentration, 200 µg/mL); (D) microparticles after three washes with fXII added (final concentration, 22 nM); buffer; buffer with 22 nM fXII added. The C and D diagrams present means ± se for 7 donors.

A more straightforward way to determine the nature of activating microparticles is flow cytometry. PFP obtained from healthy donors contained 270±160 PS+ microparticles/µl, 140±110 of them with platelet origin (CD61+) (n = 7). Microparticles concentrations were measured for 7 patients with SC (Table S4). Five of them (patients with hereditary hemolytic anemia, sepsis, hemophilia A (24 hours after hip joint surgery), multiple myeloma and pneumonia, or lymphogranulomatosis during chemotherapy) had increased microparticles amounts, the means being 2070±620 PS+, 1220±720 CD61+ microparticles/µl. Two subjects (bacterial endocarditis, hemophilia A during surgery) had normal microparticle amounts: 390 and 120 PS+, 230 and 80 CD61+ microparticles/µl, respectively. Figure 7 shows representative dot plots of microparticle samples from normal donor (A) and patient plasmas (B).

Taken together, these data indicate that microparticles play a key role in spontaneous clotting in most cases, and this role was not only in the provided phospholipid surface.

### Circulating Microparticles-induced Activation of the Contact Pathway

There are indications that activated platelets and microparticles can activate plasma through the contact pathway [26]–[28]. We aimed to find out whether microparticles circulating in plasma can induce SC in the thrombodynamics test. To do so, we carried out thrombodynamics experiments in a group of normal donors using PFP and microparticles-enriched plasma (the lower 30% after centrifugation for 30 minutes at 16000 g), and also assessed contact pathway factor activity in samples with isolated microparticles and citrate plasma diluted 50-fold as a source of fXII and its co-factors. In most cases, SC appear in microparticle-enriched healthy donor plasma (Fig. 2A).

We studied ability of circultating microparticles to activate clotting with a chromogenic assay for contact pathway factors on plasma samples from 7 donors. The substrate cleavage rate significantly (p<0.05) increased 2.9±0.5 fold after addition of microparticles to the diluted donors’ microparticle poor plasma. CTI addition significantly decreased microparticles effect (Fig. 7B). Addition of 22 nM of pure fXII to the sample with washed microparticles increased the substrate cleavage rate by an order of 1.7±0.2 compared with the microparticls-only sample (Fig. 7C). The presented data show that the circulating microparticles activate plasma coagulation through the contact pathway, which could contribute to the SC phenomenon.

## Discussion

The main result of this study is the demonstration of spontaneous clotting in plasma of patients with hematology and cardiology disorders and identification of the underlying mechanism, which in most cases is associated with circulating, contact pathway-activating procoagulant microparticles, and with circulating factors fIXa and fXIa.

These three components were to some degree implicated as a source of circulating procoagulant activity before. FXIa was found to be elevated in some diseases [1], [2], [29], as well as fIXa [29]. Increased circulating microparticles are also an established prothrombotic marker [7]–[11], [30], and there are data that their effect is not limited to extrinsic pathway stimulation or supply of phospholipid for tenase and prothrombinase. In [9], [19], [27] it was shown that natural microparticles from patients’ plasma increase thrombin generation in a fXI-dependent manner. But it was not obvious if microparticles really activate coagulation or they just accelerate reactions. Microparticles produced in vitro by platelets and erythrocytes stimulated with calcium ionophore were directly shown to activate fXII [28]. But this cell activation method is not physiological and it is known that microparticles' properties depend on activation type. This work is the first to demonstrate contact activation by natural microparticals from plasma in system of pure proteins and to confirm its significance in a global coagulation test. Here is also cleared up some important aspects of a new method that allows estimating procoagulant factors contributions to activation and propagation phase of coagulation in one simple assay.

The data of the present study show that SC is a reproducible and persistent phenomenon if all pre-analytical conditions are correct. SC may appear as a result of inappropriate blood drawing procedures, when damaged tissues contaminate the test tube or partial hemolysis happens. Long extracorporeal pre-analytical times and jerky transportation can also result in platelet vesiculation [31] and development of SC. However, SC also occurs in specimens when no preparation mistakes are made and the cause is in the plasma itself. Reliability of the phenomenon was also confirmed by the fact that SC appeared repeatedly in 16 out of 29 patients on long-term monitoring, and also by the fact that SC appear significantly more frequently in patients' plasma than in healthy donors samples. SC were reproducible in replicates, with freezing and thawing, and were still observed after the plasma was stored for longer than 3 hours.

In this study, we chose a “broad front approach” using plasmas obtained from a large number of patients suffering from various disorders. While this approach has advantages in ensuring that the phenomenon under study is ubiquitous, the statistics obtained for each specific hematological disorder was not large. Additional studies will be required to better elucidate the mechanism of SC formation in the plasmas of different patients. Similarity of the results obtained in different patients indicates that the key pathological mechanisms are very similar in different patients.

In particular, extrinsic-pathway activation was rarely observed as a cause of SC in the present study. In standard thrombodynamics assay we use CTI to inhibit contact activation that always persists in in vitro experiment. Inhibition of contact activation at the fXII level decrease SC suffisiently (data not shown), but it did not lead to complete elimination of spontaneous clotting either. This may be explained by the fact that the inhibitor used does not suppress the activation completely, or fXIIa may have enough time to produce fXIa and fIXa before addition of CTI.

FXIa and fIXa could be produced not just during the sample preparation in vitro but in patient plasma as well, as literature sources demonstrate [1], [3]–[5], [29]. These active factors may thus be viewed as an independent way to activate clotting. Their role in spontaneous clotting is supported by the fact that, in contrast to the other long-living plasma factors, fVIIa and fVa, addition of fIXa and fXIa to normal donor plasma is followed by formation of isolated spontaneous clot similar to those observed in the patient plasma. On the other hand, spontaneous clots with normal plasma containing added fXIa and, particularly, fIXa were less stable with passing time, as compared with spontaneous clotting in patient plasma. These data show that the presence of fIXa or fXIa alone does not always suffice to explain most patient cases. In the rare patient cases where we see uniform background clotting, this may be caused by a presence of fVIIa, fVa, soluble TF in the plasma.

Spontaneous clot formation in the form of isolated centers, both in plasmas of most patients and in an experimental model of normal plasma supplemented with fXIa and fIXa, indicates that the plasma contains particles that are either potent accelerators of the activation reaction or clotting activators themselves. Phospholipid microparticles may naturally serve as such particles. Additional centrifugation of PFP leading to precipitation of the microparticles results in either elimination of spontaneous clotting, or considerable reduction of clots. Clot growth rate also slows down, as a result of phospholipid surface deficiency. If the phospholipid surface is replenished with artificial vesicles, the activator clot growth rate is recovered; however, no recovery of spontaneous clotting was observed in 17 out of 22 subjects. In the instances where phospholipid replenishment led to recovery of spontaneous clotting, it is likely that clotting was mostly induced by the component dissolved in the plasma, because artificial phospholipid vesicles are unable of activating clotting themselves, rather only accelerating this process. This conclusion is also confirmed by flow cytometry results. We found a higher-than-normal quantity of annexin-positive particles in 5 from 7 patients with spontaneous clotting in the Thrombodynamics assay. A direct determination of the microparticles’ ability to activate contact-pathway clotting provided evidence that even microparticles obtained from healthy donors have this capability. These data are in agreement with publications [9], [27], [28], which demonstrated the ability of microparticles to activate thrombin generation via contact pathway. We did not investigate in our studies the origin of these microparticles in detail, but it was extremely interesting that even healthy donor microparticles could do it. In a place of damage with presence of activated platelets considerable influence of microparticles on clotting is unlikely though the question has not been studied yet. But there is risk that microparticles can activate clotting without injuiry. The acceleration of coagulation reactions due to PS on microparticles’ surface can be of the same significance for hypercoagulation state as their ability to activate coagulation directly. One can presume that normally this process is compensated with inhibitors, and only a change in the microparticle quantity or quality (possibly in a combination with factors that may be produced by the same microparticles) leads to its realization.

In this preliminary study, we did not look for direct association between spontaneous clotting and thrombosis. It is quite probably that even significant tendency for spontaneous clotting can be controlled in vivo by anti-coagulation systems of the organism. However, this is a rather direct functional test, and spontaneous clotting indicates clear abnormalities in these patients with that have conditions known to be associated with a risk of thrombosis. The obtained data demonstrate the possible importance of this phenomenon for understanding the mechanisms of hypercoagulation and may be of diagnostic importance.

## Supporting Information

Table S1 **Diagnoses, ages, and coagulation tests data of subjects whose plasma were used in Thrombodinamics test with factors IXa, XIa, TF inhibitors (subjects 11–18) or TF inhibitor only (subjects 1–23).**
(XLSX)

Table S2 **Diagnoses, ages, and coagulation tests data of subjects whose plasma were used in Thrombin generation test in platelet free plasma and microparticles rich plasma.**
(XLSX)

Table S3 **Diagnoses, ages, and coagulation tests data of subjects whose plasma were used in Thrombodinamics test in platelet free plasma, microparticles poor plasma and microparticles poor plasma supplemented with artificial phospholipid vesicles.**
(XLSX)

Table S4 **Diagnoses, ages, and coagulation tests data of subjects whose plasma were used for microparticles concentration measurement using flow cytometry.**
(XLSX)
